# A Scoping Review of Measurement Tools Evaluating Awareness and Disease-Related Knowledge in Peripheral Arterial Disease Patients

**DOI:** 10.3390/jcm13010107

**Published:** 2023-12-24

**Authors:** Carolina Machado de Melo Felix, Danielle Aparecida Gomes Pereira, Maureen Pakosh, Lilian Pinto da Silva, Gabriela Lima de Melo Ghisi

**Affiliations:** 1 Graduate Program in Rehabilitation Sciences, Department of Physiotherapy, Universidade Federal de Minas Gerais, Belo Horizonte 31270-901, Brazil; carolmmfelix91@gmail.com (C.M.d.M.F.); danielleufmg@gmail.com (D.A.G.P.); 2 Library & Information Services, Toronto Rehabilitation Institute, University Health Network, Toronto, ON M5G 2A2, Canada; maureen.pakosh@uhn.ca; 3 Graduate Program in Rehabilitation Sciences and Physical Functional Performance, Faculty of Physiotherapy, Universidade Federal de Juiz de Fora, Juiz de Fora 36036-900, Brazil; lilian.pinto@ufjf.br; 4 KITE Research Institute, University Health Network, Toronto, ON M4G 1R7, Canada

**Keywords:** awareness, patient education as topic, knowledge, peripheral artery disease, surveys and questionnaires

## Abstract

Background: Peripheral arterial disease (PAD) is the third leading cause of atherosclerotic cardiovascular morbidity worldwide, with high prevalence and associated complications, and is often overlooked and undertreated. Research has shown that there is a profound lack of PAD-related knowledge and awareness; additionally, information sources are not often reliable and accessible. The objective of this scoping review was: (1) to identify and critically appraise instruments that measure patients’ disease-related knowledge/awareness about PAD, and (2) to characterize the current state of knowledge/awareness levels among these patients. Methods: This systematic review was conducted and reported in accordance with the PRISMA statement. Six databases (APA PsycInfo, CINAHL Ultimate, Embase, Emcare Nursing, Medline ALL and Web of Science Core Collection) were searched, and search strategies were developed utilizing the PICO framework. Potential studies of any methodological design were considered for inclusion through a snowball hand search. Data from the included articles were extracted by a reviewer, and the extraction accuracy was independently cross-checked by another author. Results: The initial database search yielded 9832 records, of which sixteen studies (thirteen quantitative and three qualitative) were included. Only three questionnaires had their psychometric properties assessed. Questionnaire items focused on the following topics: definition/characteristics, risk factors/causes, treatment, complications, and personal issues regarding the perception/management of the disease. Overall, knowledge/awareness about PAD was low among patients. Conclusions: This study identified major gaps in PAD education, including the lack of availability of a validated measurement tool addressing all educational topics relevant to care and low knowledge/awareness of patients about their condition.

## 1. Introduction

Peripheral arterial disease (PAD)—a chronic arterial occlusive disease of the lower extremities—is a powerful predictor for all-cause, cardiovascular, and cerebrovascular morbidity and mortality, especially in older adults [1,2,3]. It is the third leading cause of atherosclerotic cardiovascular morbidity worldwide, affecting approximately 202 million people [1]. Despite the high prevalence and associated complications, PAD is often overlooked and undertreated [4]. Research has shown there is a profound lack of PAD-related knowledge and awareness in the general population [5,6,7], as well as healthcare providers [8,9,10] and those diagnosed with this condition [11]. Additionally, information sources for PAD are often not reliable and accessible [12,13].

Patient education is essential in the care of those living with cardiovascular diseases (CVD) [14,15], including PAD [3,16]. Patients require information about responding to symptoms, risk factors, and how to self-manage their disease to reduce this excess risk [14]. Indeed, patient education leads to not only increased knowledge but increased physical activity, improved nutrition, tobacco cessation, and medication adherence, as well as reductions in anxiety and depressive symptoms [14,15]. Delays in the presentation and appropriate management of the condition may lead to complications such as ischaemia, amputation, and death [4]. Research shows that educating and supporting patients with CVD leads to a better understanding of their central role in disease management, making informed decisions about their care, and engaging in heart-healthy behaviours [17]. Many strategies are used by healthcare providers to educate their patients, from discharge education [18] to structured programs following an event [19]. However, improvements in patients’ disease-related knowledge and awareness following educational interventions are not always achieved, which can compromise patients’ outcomes and the progression of their disease [20,21,22]. In this context, identifying patients’ needs and knowledge gaps is important to guide healthcare providers in supporting their patients’ learning journey.

Since the global prevalence of PAD is increasing [23] and prior studies have demonstrated not only a lack of knowledge and awareness about PAD in multiple groups—including those living with this disease—but a lack of reliable information sources, there is an urgent need to develop educational strategies centred on the needs of patients with PAD to support shared decision-making and self-management. As a first step in this process, it is important to understand patients’ knowledge levels and gaps; thus, a valid and reliable instrument to measure disease-related knowledge and awareness of PAD is needed. Therefore, the objective of this scoping review was: (1) to identify and critically appraise instruments that measure patients’ disease-related knowledge/awareness about PAD; and (2) to characterize the current state of knowledge/awareness levels among these patients.

## 2. Materials and Methods

Methods for this systematic review were based on the Cochrane Handbook for Systematic Reviews of Interventions [24]. The review was conducted in accordance with the Preferred Reporting Items for Systematic Reviews and Meta-Analyses (PRISMA) 2020 guidelines [25]. The protocol for this systematic review was registered prospectively on Open Science Framework (OSF; registration: https://osf.io/chzwa/, accessed on 15 December 2023).

### 2.1. Eligibility Criteria

The inclusion criteria involved studies of any methodological design assessing disease-related knowledge or the awareness of populations with PAD or intermittent claudication. Narrative, systematic, and scoping reviews were considered a source of additional primary studies. Non-peer-reviewed literature was excluded. Studies published in any language were considered.

### 2.2. Information Sources and Search Strategy

The following databases were searched from inception to January 2023: APA PsycInfo (Ovid), CINAHL Ultimate (EBSCOhost), Embase (Ovid), Emcare Nursing (Ovid), Medline ALL (Ovid; includes PubMed non-Medline records), and Web of Science Core Collection. The search strategies were developed in collaboration with an Information Specialist, utilizing the PICO framework, subject headings as appropriate for each database, and free-text terms relevant to the topical concepts. Potential studies were considered for inclusion through a snowball hand search. The full search strategy is available in the Supplementary File S1.

### 2.3. Selection Process

After the literature search was performed, all identified studies were uploaded into Covidence (Veritas Health Innovation, Melbourne, Australia), and duplicate citations were removed. After training and calibration, two researchers (CMMF; GLMG) independently screened all abstracts identified by the search strategy for inclusion. The full-text of potentially-eligible citations were obtained and assessed independently for eligibility by the two researchers. The degree of agreement between the two reviewers was very high, with an intraclass correlation coefficient of 0.89 (95% CI: 0.896–1.000) across the entire evaluation. Any disagreements were resolved by discussion or by consultation with another author (DAGP), where agreement could not be reached.

### 2.4. Data Extraction and Synthesis

Data from included articles (i.e., study design, sample, setting, measurement tools and outcomes reported were extracted from included studies) were extracted by the first author (CMMF) and independently cross-checked by another author (DAGP) to secure accuracy. All available data—including figures, tables, and supplementary materials—were considered for data synthesis. Study results were synthesized in a tabular format following the Synthesis Without Meta-analysis (SWiM) reporting guideline [26], and a formal narrative synthesis was used to analyse outcomes as they could not be meta-analysed due to data heterogeneity. The COnsensus-based Standards for the selection of health Measurement INstruments (COSMIN) was used to critically appraise identified instruments that measure patients’ disease-related knowledge/awareness about PAD [27].

The quality of the included studies was assessed using the five-item Mixed-Methods Assessment Tool (MMAT) [28], which applies to multiple study designs (Supplementary File S2). Each item is rated as being present (yes), not present (no), or indeterminant (unclear). The total score ranges from 0 to 5, with scores of 4 and 5 indicating “high” quality.

## 3. Results

### 3.1. Study Selection

**Figure 1:**
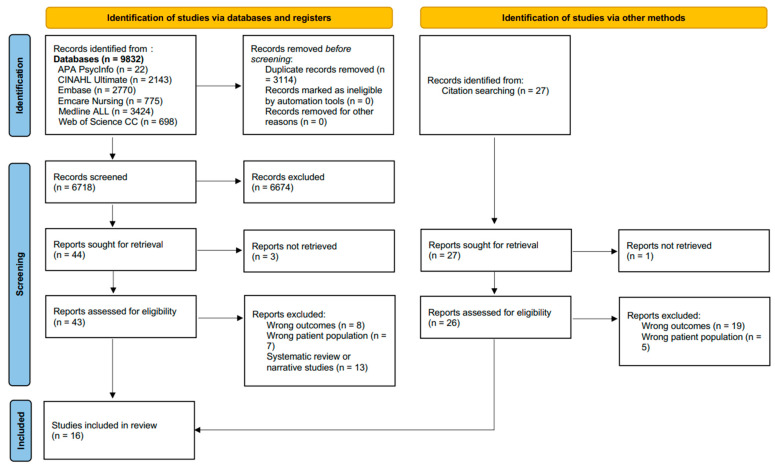
PRISMA flow diagram.

The initial database search yielded 9832 records. After excluding duplicates and studies that did not meet eligibility criteria, 43 full-text articles were assessed for eligibility. Overall, 16 studies were included in this review. Figure 1 presents the PRISMA flow diagram.

### 3.2. Study Characteristics

Table 1 summarizes the characteristics of the included studies. Of the 16 studies included in the review, 13 were quantitative (12 cross-sectional and 1 prospective cohort study) [11,17,29,30,31,32,33,34,35,36,37,38,39] and three were qualitative in design [21,22,40]. Two were abstract-only publications [35,38]. Eleven studies were conducted in the European Region [17,21,22,29,30,31,32,33,38,40], three in the Region of the Americas [11,35,39] and two in the South-East Asian Region [34,37]. Except for one study published in Korean [26] and one in Spanish [36], all others were published in English. As shown in Table 1, the first study was published in 2003 [39].

Data from the qualitative studies were collected using focus groups (*n* = 2) [22,40] and semi-structured interviews (*n* = 1) [21]. Data from all 13 quantitative studies were collected using questionnaires. When reported (*n* = 8), the number of questions in these questionnaires ranged from 5 to 44 items (median = 17). The questionnaires used multiple-choice questions, correct/incorrect questions, Likert-scale, correct/incorrect, true/false, and a combination of questions according to Table 1.

Sample sizes across the included studies ranged from 19 to 797, with a median of 106 participants; overall, there were 2408 PAD patients included. In the majority of the studies (81.2%), the sample was comprised of mostly male participants (i.e., >50%). The mean age of participants ranged from 61 to 72 years old. Only three studies reported the ethnicity of participants [11,39,40], of which the majority were Caucasian (Table 1).

The quality of each study was assessed using the MMAT and results are shown in Table 1. Overall, four (25.0%) of the included studies presented scores higher than 4 [28]. The median number of ‘yes’ responses (indicating good quality) per study was three out of five.

### 3.3. Critical Appraisal of Identified Instruments

Of the 13 studies that used questionnaires to assess the knowledge and awareness of PAD patients, three (23.1%) evaluated measurement properties: content validity [37], reproducibility [37], and internal consistency [33,36]. Following COSMIN, all properties evaluated were considered acceptable (Table 2).

### 3.4. Measurement of Disease-Related Knowledge and Awareness in PAD Patients

**Figure 2:**
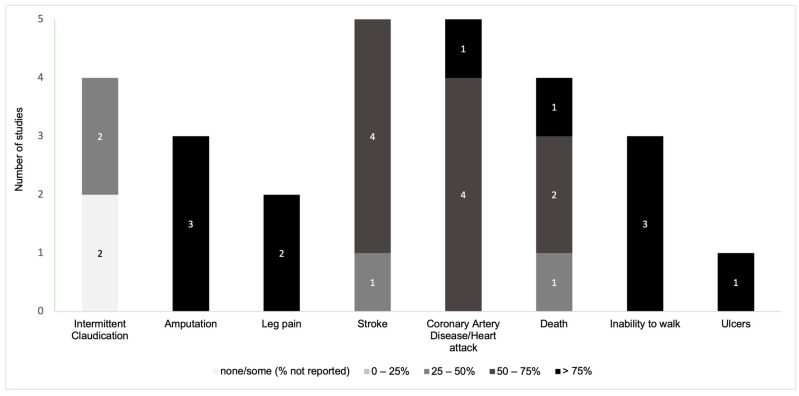
Distribution of the knowledge and awareness about signs/symptoms of PAD evaluated in studies.

**Figure 3:**
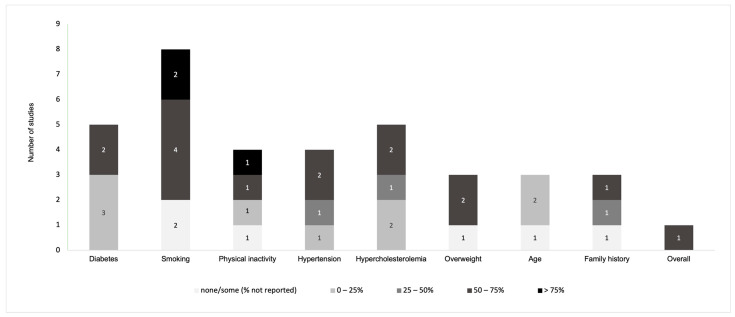
Distribution of the knowledge and awareness about risk factors of PAD evaluated in studies.

**Figure 4:**
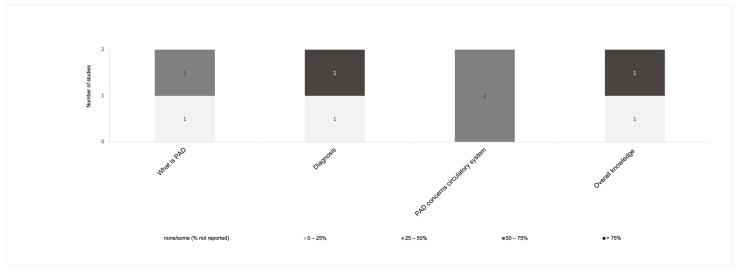
Distribution of the knowledge and awareness about the pathophysiology of PAD evaluated in studies.

**Figure 5:**
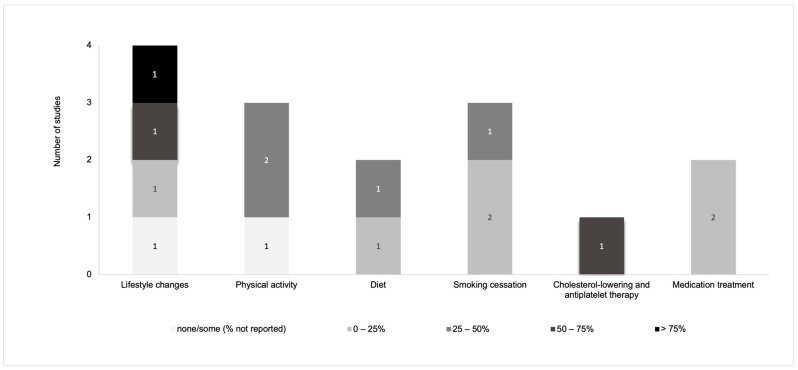
Distribution of the knowledge of, and awareness about, management and pharmacological therapy of PAD evaluated in studies.

Overall, disease-related knowledge and awareness in PAD patients were measured with regard to signs/symptoms and complications in twelve instruments (Figure 2) [11,21,22,29,31,32,33,36,37,38,39,40], risk factors in nine instruments (Figure 3) [11,21,22,29,30,31,36,38,40], pathophysiology in eight instruments (Figure 4) [11,29,31,32,36,37,38,40], and management and pharmacological therapy in six instruments (Figure 5) [31,35,36,38,39,40]. One of the studies presented general knowledge without describing specific areas [34]. None of the identified instruments included questions from all these knowledge areas. Supplementary File S3 includes the data used to create Figure 2, Figure 3, Figure 4 and Figure 5 regarding the percentage of participants that selected correct answers by topic, which was divided into quartiles (0–25%, 25–50%, 50–75%, and >75%).

Among PAD signs/symptoms, four studies included knowledge questions about intermittent claudication. Of these, two studies reported the percentage of participants who identified intermittent claudication as a PAD sign or symptom (21.0% and 47.1%) [21,36] and two studies showed that patients did not effectively recognize intermittent claudication as a symptom of PAD [22,40]. Knowledge about other complications related to PAD was also included as the following: coronary artery disease/heart attack (*n* = 5 studies; median % of patients who identified this PAD complication = 65.7%; range = 58.8% to 77.0%) [11,29,31,33,38], stroke (*n* = 5; median % = 58.8%; range = 28.7% to 71.0%) [11,29,33,38,39], death (*n* = 4; median = 59.1%; range = 26.6% to 84.0%) [11,29,33,39], inability to walk (*n* = 3; median = 84.0%; range = 80.0% to 85.7%) [11,29,33], amputation (*n* = 3; median = 78.0%; range = 73.2% to 91.0%) [11,29,33], leg pain (*n* = 2; median = 86.4%; range = 85.7% to 87.0%) [11,33], and ulcers (*n* = 1; 76.0% participants identified this PAD complication) [33].

Among knowledge about risk factors, smoking was most commonly assessed (*n* = 6) with a median of 68.6% of participants identifying smoking as a PAD risk factor (range = 52.0% to 86.0%) [11,21,29,30,31,38]; two studies did not describe the percentage [22,40]. Knowledge about other PAD risk factors was also assessed as follows: diabetes (*n* = 5 studies; median % of participants who identified diabetes as a PAD risk factor = 21.6%; range = 8.0% to 63.2%) [11,29,30,31,38]; hypercholesterolemia (*n* = 5; median = 30.0%; range = 12.0% to 73.0%) [11,29,30,31]; physical inactivity (*n* = 3; median = 73.0%; range = 23.0% to 75.7%) [11,29,31] and one study did not describe the percentage [40]; hypertension (*n* = 4; median = 45.4%; range = 11.4% to 69.0%) [11,29,30,38]; being overweight (*n* = 2; median = 63.1%; range = 55.2% to 71.0%) [11,29], and one study did not describe the percentage [40]; age (*n* = 2; median = 12.9%; range = 4.0% to 21.7%) [31,38] and one without percentage [40]; and family history (*n* = 2; median = 50.9%; range = 36.8% to 65%) [11,29] and one without percentage [40].

Regarding the pathophysiology of PAD, two studies [29,40] investigated “what is PAD”, with 32.0% of participants knowing the response in one study [29]; the other study did not describe knowledge percentages [40]. Considering PAD diagnosis, 64.2% of respondents were aware of it in one study [11]. Two studies identified knowledge about dysfunctions in the circulatory system related to PAD, with knowledge percentages ranging from 21.0% to 26.0% [31,38].

Knowledge about PAD management varied across studies. Knowledge about “managing PAD for the rest of their lives/lifestyle” was evaluated in three studies, with a median of correct answers of 57.2% (range = 13.0% to 81.0%) [31,35,36]. In addition, one study reported that “most participants were aware that there were things they could do by themselves to manage their health condition” [40], but no extra information was reported. Knowledge about quitting smoking was also evaluated in three studies (median correct answers = 24.0%; range = 20.0% to 30%) [31,35,38]. Knowledge about self-management and adherence to health behaviours was assessed in terms of physical activity in two studies, with knowledge ranging from 35.0% to 48.0% [31,38]. Knowledge about following a healthy diet as an important tool in PAD management was reported in two studies, with a knowledge range of 10.0% to 32.0% [31,38].

Finally, four studies assessed knowledge about pharmacological therapy [31,36,38,39]. Of these, one study assessed overall knowledge about pharmacological therapy, with 65.0% of participants having knowledge about it [36]. In addition, two studies assessed knowledge about the importance of medication in the treatment of PAD (knowledge range = 4.0% to 12.0%) [31,38], and one study assessed knowledge about cholesterol-lowering therapy and antiplatelet therapy, with 75.8% of participants identifying knowledge about this [39].

**Figure 6:**
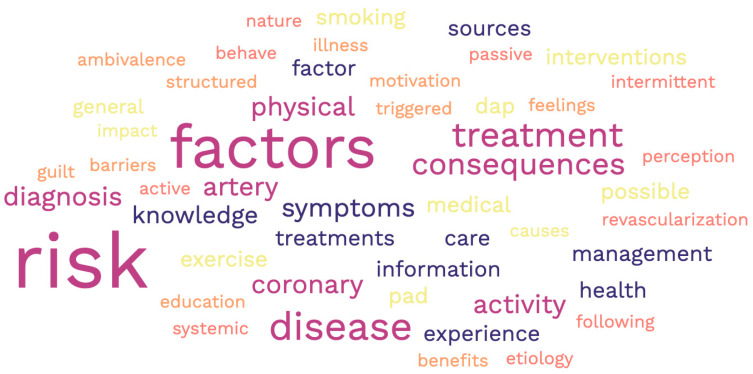
Word cloud of most common terms included in the knowledge/awareness questions.

The word cloud illustrates the most common terms in the questions and statements that are part of the included questionnaires in this review. Risk factors, complications of PAD, and treatment were the terms found most frequently in these instruments (Figure 6).

## 4. Discussion

The prevalence of PAD has dramatically increased globally, calling for efforts to improve knowledge and awareness about this disease. There is a paucity of research published on education about PAD [3,16], which this scoping review aimed to address. Through our work, 13 questionnaires that measured patients’ disease-related knowledge/awareness about PAD were identified. However, few or no measurement properties were evaluated, which limits their use and the overall validity of their results. Participants in the included studies had knowledge associated with smoking, physical inactivity, and being overweight as risks for their disease and its general complications; however, knowledge inadequacy was generally observed in important topics, such as the management of PAD. Taking these findings together underscores the need for more research and efforts to educate PAD patients.

PAD has long been underdiagnosed and it is estimated that up to half of all people with PAD are undetected [4]. Many systematic reviews have focused on this disease, including understanding patients’ beliefs about their illness [41], addressing the importance of socioeconomic determinants of health in the care of PAD [42], and evaluating what the public and healthcare practitioners and trainees know about PAD [8]. Although these are all important research questions, to initiate timely treatment, those living with PAD must first recognize their symptoms and seek medical attention. This is only possible when they are aware of or understand their condition, which was the focus of this first review of publications addressing the identification and critical appraisal of instruments that measure patients’ disease-related knowledge/awareness about PAD and characterizing the current state of knowledge/awareness levels among them.

Assessing patients’ knowledge about PAD is considered a first step to implementing educational interventions aiming to optimize PAD self-management [3]. When the healthcare team is aware of their patients’ knowledge gaps, they can target educational strategies and bridge such gaps. Educating patients in a patient-centred manner is associated with better self-care, which can ultimately improve outcomes and promote a better quality of life. The use of questionnaires in clinical practice can guide these health professionals in the care and education of their patients in a practical and low-cost way [43]. Questionnaires should be developed, and measurement properties should be tested, following a rigorous process [27] to confirm its validity. Not assessing psychometric properties implies tools are not reliable for the proposed outcome, and therefore are unable to effectively measure their proposed outcomes in clinical practice [27]. There is an urgent need to develop and psychometrically validate a questionnaire to assess disease-related knowledge about PAD that encompasses multiple areas, including pathophysiology, signs, symptoms, risk factors, complications, management, and treatments.

Disease-related knowledge is a challenging construct to evaluate, given it is influenced by multi-level factors, such as environment, socioeconomic status, culture, and health literacy [44,45]. Overall, important topics related to PAD were included (e.g., aetiology, symptoms, risk factors, complications, treatment, and management). However, we were unable to identify one single instrument that combined all of these important knowledge areas, which is typically present in validated questionnaires designed to assess knowledge of other cardiovascular conditions [46,47,48].

Despite the overall higher percentage of correct answers for risk factors—which indicate higher knowledge–patients with PAD often do not understand risk factors and often do not believe that lifestyle interventions will make a difference in disease outcomes, thus making it difficult to change behaviour [2,49]. Addressing patient knowledge gaps and uncertainty surrounding the disease process is critical to driving behaviour change [40]. In addition, the lack of understanding about the aetiology and nature of intermittent claudication (i.e., the main symptom of PAD) makes it difficult to change one’s lifestyle and make decisions, which reinforces the importance of a better understanding of the disease through educational strategies [21].

Low knowledge about PAD seems to be common across multiple groups. Findings from studies that assessed PAD knowledge in medical students and healthcare professionals showed poor overall knowledge [10,12,13]. Studies have also shown that the general population is poorly informed about PAD, with significant knowledge gaps regarding its causes, risk factors, and complications [5,6,7]. Lack of knowledge from healthcare teams and the general public may lead to missed opportunities for disease recognition and diagnosis [5,6,7]. Alternatively, health professionals may incorrectly attribute symptoms to existing conditions [9,10,12], which can postpone patients receiving the right care. Efforts to increase PAD knowledge should not be limited to patients but should be expanded to other groups and this is also true for measurement tools: assessing knowledge and identifying gaps can provide important insights into PAD recognition and awareness, and stimulate the development of strategies to educate multiple groups [3].

Cardiovascular rehabilitation (CR) is an outpatient model of secondary prevention care that can mitigate the burden of CVDs [50]. CR participation reduces cardiovascular morbidity and mortality by 20% [51]. Clinical practice guidelines highlight the importance of this intervention in the care of people living with CVDs, including PAD [52,53]. Patient education is a core component of CR programs [50] and a quality indicator [54]. This review highlights a knowledge gap related to the education of PAD patients (i.e., knowledge inadequacy was generally observed among study participants and few or no measurement properties were evaluated in questionnaires that measured patients’ disease-related knowledge/awareness about PAD), which affects CR delivery. More research around this area will also support the delivery of CR for those living with PAD.

Our study has some limitations. First, a meta-analysis was performed due to the heterogeneity in the design and outcome measures of included studies. Second, most studies were quantitative and non-randomized; therefore, results were based on a less rigorous method. Third, included studies were conducted mainly in higher income settings and reported in English, making it difficult to generalize findings to other regions and languages. Fourth, only three instruments evaluated psychometric properties [33,36,37], and few measurements were assessed among them. We were unable to identify one instrument that could be used in clinical practice, as no implementation information was provided. Future research should follow a rigorous process for developing and psychometrically validating an instrument to assess disease-related knowledge and awareness of PAD patients. Translation and cross-cultural adaptation of this instrument to reach multiple cultural groups is also warranted.

## 5. Conclusions

This study identified major gaps in PAD education, including the unavailability of a validated measurement tool addressing all educational topics relevant to care and low knowledge/awareness of patients about their condition. Future studies should be carried out to develop and psychometrically validate such instruments, which could ultimately improve clinical practice by understanding knowledge gaps and informing providers of their patients’ information needs. This would ultimately increase patients’ knowledge about PAD, which could lead to disease management and engaging in heart-healthy behaviours.

## Figures and Tables

**Table 1:** Characteristics of included studies (*n* = 16).

Author Year Country	Study Design Participants Setting Quality	Sample Size Mean Age (SD or Range) % Male Ethnicity	Method Used for Outcomes Measurement
Wann-Hansson and Wennick ♦ 2016 [22] Sweden	Qualitative Patients with PAD undergoing vascular intervention Vascular centre 5/5	21 70.0 (range 50–81) years old 42.9% male NR	Focus groups Themes: Knowledge about vascular disease, risk factors and treatment
Gorely et al. ♦ 2015 [40] United Kingdom	Qualitative Patients with PAD and IC Hospital 5/5	24 71.0 (8.0) years old 71.0% male Caucasian (100%)	Focus groups Themes: experiences of living with PAD/IC, attitudes and beliefs about PAD/IC, educational needs of people with PAD/IC, attitudes and beliefs about physical activity/exercise, levers and barriers for exercise behaviour change, opinions of behaviour change interventions, particularly structured education
Lokin et al. ♦ 2015 [21] The Netherlands	Qualitative Patients with PAD and IC Multiple Hospital 5/5	19 60.8 (6.7) years old 52.6% male NR	Semi structured individual interviews Themes: Knowledge about disease, aetiology and lifestyle
Keelan et al. ♦ 2021 [29] Ireland	Cross-sectional VC, CVC and the ED patients Multiple Hospital 3/5	49 67.1 (9.7) years old 59.0% male NR	Questionnaire (structure NR) Number of items NR Awareness about disease entity, PAD risk factors and potential consequences of PAD if left untreated
Byskosh et al. ♦ 2022 [11] United States	Cross-sectional Patients with PAD Vascular surgery clinic 3/5	109 69.4 (11.0) years old 60.6% male Caucasian 56.9%/African American 34.9%/Other 8.3%	Questionnaire (multiple-choice questions) 44 items (score range 0–100%) Knowledge about general risk factors and potential consequences of PAD, awareness of own medical history and PAD education preferences.
Udelnow et al. ♦ 2020 [32] Germany	Prospective cohort study Patients with PAD undergoing vascular intervention Hospital 3/5	198 NR NR NR	Questionnaire (correct/incorrect) 20 items (score range 0–20) knowledge about the disease, self-information, smoking habits, and treatment expectations
Bolt et al. ♦ 2020 [17] The Netherlands	Cross-sectional survey Patients with PAD Medical centre 2/5	108 72.0 (range 53–92) years old 57.0% male NR	Questionnaire (5-point Likert-scale) 17 items (score range NR) 10 items regarding: knowledge (19 a/b, 20), risk perception (21 a/b/c), attitude (22/23), self-efficacy (24) and intention towards physical exercise (25).
Builyte et al. ♦ 2019 [33] Denmark	Cross-sectional Patients PAD or Coronary Artery Disease Hospital 4/5	63 PAD 68.3 (11.8) years old 71.0% male NR	Questionnaire (multiple-choice questions) 14 items (score range NR) knowledge about of risk factors for PAD and Coronary Artery Disease, consequences, severity and other non-vascular illnesses
El Jang et al. # 2018 [34] Korea	Cross-sectional Patients with PAD Hospital and outpatient clinic 3/5	104 66.4 (13.3) years old 92.3% male NR	Questionnaire (correct/incorrect/‘I don’t know’) 15 items (score range 0–15) knowledge about definition and characteristics of PAD, management (exercise, diet, smoking cessation), treatment, and risk factors
Provance et al. * 2018 [35] United States	Cross-sectional Patients with PAD and IC Vascular clinics 0/5	797 69.2 (8.7) years old 55.2% male NR	Questionnaire (correct, incorrect, and “not sure”) Items NR Knowledge about PAD treatment options
Martínez et al. + 2017 [36] Spain	Cross-sectional Post-Angiology and Vascular patients. Hospital 3/5	120 72.0 (13.0) years old 79.0% male NR	Questionnaire (true/false) 24 items (score range 0–24) Knowledge about disease, risk factors, therapeutic regimen, pharmacotherapy and warning signs
Vasaroangrong et al. ♦ 2016 [37] Thailand	Cross-sectional Patients with PAD Outpatient clinic 2/5	212 66.0 (12.6) years old 59.9% male NR	Questionnaire (yes/no) 16 items (score range NR) Knowledge about PAD symptoms (seven items), risk factors (five items), and the effects of PAD (four items)
Owens et al. * 2013 [38] Ireland	Cross-sectional Patients with PAD Hospital 0/5	97 Age distribution (years): 18–34 (3.0%); 35–54 (4.0%); 45–54 (7.1%); 55–65 (26.3%); 65–74 (37.0%); >75 (22.2%) 54.6% male NR	Questionnaire (NR) Items NR Knowledge about understanding of peripheral vascular disease, risk factors, health improvement strategies, exercise and risk of other vascular disease
Coughlin et al. ♦ 2007 [30] United Kingdom	Cross-sectional Patients with PAD Vascular surgery department 3/5	70 72.0 (range 42–89) years old 70.0% male NR	Questionnaire (open and multiple-choice questions) 5 items (score range NR) Knowledge about risk factors and prevention/treatment
Willigendael et al. ♦ 2004 [31] The Netherlands	Cross-sectional Patients with PAD and general population Netherlands database 3/5	281 Age distribution (years): 35–44 (7.0%) 45–54 (19.0%) 55–64 (23.0%) > 64 (51.0%) 49.0% male NR	Questionnaire (open and multiple-choice questions) Items NR Knowledge about risk factors, medical advice and treatment
McDermott et al. ♦ 2003 [39] United States	Cross-sectional Patients with PAD or Coronary Artery Disease or no disease Vascular Laboratories and Vascular Centre 2/5	136 70.2 (8.1) years old 52.9% male African American: 15.4%	Questionnaire (5-point Likert-scale) 3 sections (score range NR) Knowledge about health behaviour regarding CVD prevention; risk of heart disease, stroke, and death; importance of specific CVD risk factor interventions

Legend: PAD = peripheral artery disease; IC = intermittent claudication; SD = standard deviation; NR = not reported; PTA = percutaneous transluminal angioplasty; SET = supervised exercise therapy; VC = vascular clinic patients; CVC = cardiovascular clinics patients; ED = emergency department patients; AAA = abdominal aortic aneurysm; CVD = cardiovascular disease. * Abstract ♦ English language # Korean language + Spanish language.

**Table 2:** Evaluation of psychometric properties of identified questionnaires from quantitative studies (*n* = 13).

Author (Year)	Content Validity	Internal Consistency	Criterion Validity	Construct Validity	Reproducibility Agreement	Reproducibility Reliability	Responsiveness	Floor and Ceiling Effect	Interpretability
Keelan (2021) [29]	NP	NP	NP	NP	NP	NP	NP	NP	NP
Byskosh (2022) [11]	NP	NP	NP	NP	NP	NP	NP	NP	NP
Udelnow (2020) [32]	NP	NP	NP	NP	NP	NP	NP	NP	NP
Bolt (2020) [17]	NP	NP	NP	NP	NP	NP	NP	NP	NP
Builyte (2019) [33]	NP	appropriate	NP	NP	NP	NP	NP	NP	NP
Da El et al. (2018) [34]	NA	NA	NA	NA	NA	NA	NA	NA	NA
Provance (2018) [35]	NP	NP	NP	NP	NP	NP	NP	NP	NP
Martinez (2017) [36]	NP	appropriate	NP	NP	NP	NP	NP	NP	NP
Vasaroangrong (2016) [37]	appropriate	NP	NP	NP	NP	appropriate	NP	NP	NP
Owens (2013) [38]	NP	NP	NP	NP	NP	NP	NP	NP	NP
Coughlin (2007) [30]	NP	NP	NP	NP	NP	NP	NP	NP	NP
Willigendael (2004) [31]	NP	NP	NP	NP	NP	NP	NP	NP	NP
McDermott (2003) [39]	NP	NP	NP	NP	NP	NP	NP	NP	NP

Legend: NP = Not Performed; NA = Not Applicable.

## Data Availability

Data is available upon request.

## Supplementary Materials

The following supporting information can be downloaded at: https://www.mdpi.com/article/10.3390/jcm13010107/s1, File S1: Search Strategy; File S2: Description of the quality of the analysed instruments evaluated by the Mixed-Methods Assessment Tool (MMAT); File S3: Description of the knowledge and awareness evaluated in the instruments identified.
