# Supplementary material for: A Scoping Review of Measurement Tools Evaluating Awareness and Disease-Related Knowledge in Peripheral Arterial Disease Patients
Source: J Clin Med. 2023 Dec 24;13(1):107. doi: 10.3390/jcm13010107 (PMC10780123; doi:10.3390/jcm13010107)
Supplement: Supplementary file 1 [file jcm-13-00107-s001.zip › jcm-2768981-supplementary.pdf]

Supplementary Files

S1. Search Strategy

Peripheral Artery Disease & Knowledge/Awareness Information

Search Strategy: Ovid MEDLINE(R) ALL <1946 to January 05, 2023>

----

1 [Population: Peripheral Artery Disease]

2 exp Peripheral Arterial Disease/ (10891)

3 Intermittent Claudication/ (8371)

4 (peripheral arter* adj3 (obstructi* or obliterati* or occlusi* or disease)).tw,kw. (19596)

5 (limb* adj3 (ischemia or ischaemia)).tw,kw. (12633)

6 (intermittent adj3 claudication).tw,kw. (5488)

7 or/2-6 (40307)

8 [Outcomes: Knowledge/Awareness-Related Information]

9 Patient Education as Topic/ (88167)

10 Health Knowledge, Attitudes, Practice/ (125338)

11 health education/ (63306)

12 exp consumer health information/ (12859)

13 health promotion/ (80495)

14 Awareness/ (21694)

15 exp "Surveys and Questionnaires"/ (1193443)

16 ((educat* or knowledg* or aware* or unaware* or inform* or survey* or questionnaire* or tool* or instrument* or inventor*) adj5 (peripheral arter* disease* or PAD)).tw,kw. (959)

17 (((understand* or familiar*) adj4 (patient* or public)) and (peripheral arter* or PAD)).tw,kw. (58)

18 (consequen* adj4 (PAD or peripheral arter*)).tw,kw. (105)

19 ((interview* adj3 data) and (peripheral arter* disease* or PAD)).tw,kw. (10)

20 or/9-19 (1456225)

21 7 and 20 (3535)

22 limit 21 to "humans only (removes records about animals)" (3511)

23 remove duplicates from 22 (3510)

24 23 not (Letter or Editorial or News).pt. (3424)

****

S2. Description of the quality of the analyzed instruments evaluated by the Mixed-Methods Assessment Tool (MMAT)

Author (Year)	Q1	Q2	Q3	Q4	Q5	Total/5
Gorely (2015)	Yes	Yes	Yes	Yes	Yes	5
Wann-Hansson (2016)	Yes	Yes	Yes	Yes	Yes	5
Lokin (2015)	Yes	Yes	Yes	Yes	Yes	5
Keelan (2021)	Yes	No	Yes	No	Yes	3
Byskosh (2022)	Yes	No	Yes	No	Yes	3
Udelnow (2020)	Yes	No	No	Yes	Yes	3
Bolt (2020)	Yes	No	No	No	Yes	2
Builyte (2019)	Yes	No	Yes	Yes	Yes	4
El Jang (2018)	Yes	No	No	Yes	Yes	3
Provance (2018)	Unclear	Unclear	Unclear	Unclear	Unclear	0
Martinez (2017)	Yes	No	No	Yes	Yes	3
Vasaroangrong (2016)	Yes	No	No	No	Yes	2
Owens (2013)	Unclear	Unclear	Unclear	Unclear	Unclear	0
Coughlin (2007)	Yes	Yes	No	No	Yes	3
Willigendael (2004)	Yes	No	Yes	No	Yes	3
McDermott (2003)	Yes	No	No	No	Yes	2

S3. Description of the knowledge and awareness evaluated in the instruments identified

Main knowledge topic	Subtopic	Studies assessing knowledge about the topic	Percentage of study participants that reported having knowledge/awareness about the specific topic	Code
Risk factor for PAD	Diabetes	Byskosh (2022)	63.2%	+++
		Keelan (2021)	61.0%	+++
		Owens (2013)	21.6%	+
		Coughlin (2007)	14.0%	+
		Willigendael (2004)	8.0%	+
	Smoking	Keelan (2021)	86.0%	++++
		Lokin (2015)	79.0%	++++
		Byskosh (2022)	74.3%	+++
		Owens (2013)	62.9%	+++
		Coughlin (2007)	57.0%	+++
		Willigendael (2004)	52.0%	+++
		Gorely (2015)	Widespread awareness that smoking is a risk factor for PAD	00

		Wann-Hansson (2016)	Smoking triggered feelings of guilt and ambivalence due to its harmful effect	00
	Physical inactivity	Byskosh (2022)	75.7%	++++
		Keelan (2021)	73.0%	+++
		Willigendael (2004)	23%	+
		Gorely (2015)	Uncertain about lack of exercise as a risk factor for PAD	0
	Hypertension	Keelan (2021)	69.0%	+++
		Byskosh (2022)	64.8%	+++
		Owens (2013)	26.0%	++
		Coughlin (2007)	11.4%	+
	Hypercholesterolemia	Keelan (2021)	73.0%	+++
		Byskosh (2022)	70.5%	+++
		Owens (2013)	30.0%	++
		Coughlin (2007)	17.0%	+
		Willigendael (2004)	12.0%	+
	Overweight	Keelan (2021)	71.0%	+++
		Byskosh (2022)	55.2%	+++

		Gorely (2015)	Recognized as a risk factor	00
	Age	Owens (2013)	21.7%	+
		Willigendael (2004)	4.0%	+
		Gorely (2015)	Recognized as a risk factor	00
	Family history	Keelan (2021)	65.0%	+++
		Byskosh (2022)	36.8%	++
		Gorely (2015)	Recognized as a risk factor	00
	Overall	Martinez (2017)	66.8%	+++
Signs/Symptoms of PAD	Intermittent Claudication	Martinez (2017)	47.1%	++
		Lokin (2015)	21.0%	+
		Gorely (2015)	Many were unsure what is intermittent claudication	0
		Wann-Hansson (2016)	Unaware of symptoms	0
	Overall symptoms	Vasaroangrong (2016)	Knowledge 'quite low'	0
PAD Consequences	Amputation	Builyte (2019)	91.0%	++++
		Keelan (2021)	78.0%	+++
		Byskosh (2022)	73.2%	++

	Leg pain	Builyte (2019)	87.0%	+++
		Byskosh (2022)	85.7%	++++
	Stroke	Builyte (2019)	71.0%	+++
		Byskosh (2022)	65.1%	+++
		Owens (2013)	58.8%	+++
		Keelan (2021)	55.0%	+++
		McDermott (2003)	28.7%	++
	Coronary Artery Disease/Heart attack	Willigendael (2004)	77.0%	++++
		Builyte (2019)	71.0%	+++
		Byskosh (2022)	65.7%	+++
		Keelan (2021)	59.0%	+++
		Owens (2013)	58.8%	+++
	Death	Builyte (2019)	84.0%	++++
		Byskosh (2022)	63.1%	+++
		Keelan (2021)	55.0%	+++
		McDermott (2003)	26.6%	++
	Inability to walk	Byskosh (2022)	85.7%	++++

		Builyte (2019)	84.0%	++++
		Keelan (2021)	80.0%	++++
	Ulcers	Builyte (2019)	76.0%	++++
	Overall	Udelnow (2020)	A minority of the patients remembered possible minor and major complications	0
		Vasaroangrong (2016)	Knowledge ‘quite low’	0
Pathophysiology of PAD	What PAD is	Keelan (2021)	32.0%	++
		Gorely (2015)	Unable to define PAD	0
	Diagnosis	Byskosh (2022)	64.2%	+++
		Udelnow (2020)	A minority of the patients remembered the diagnosis	0
	PAD concerns circulatory system	Owens (2013)	26.0%	++
		Willigendael (2004)	21.0%	+
	Overall knowledge about the disease	Martinez (2017)	60.5%	+++
		Vasaroangrong (2016)	Quite low	0
Management of PAD		Provance (2018)	81.0%	+++

	Manage PAD for the rest of their lives/ lifestyle changes	Martinez (2017)	57.2%	+++
		Willigendael (2004)	13.0%	+
		Gorely (2015)	Most participants were aware that there were things they could do themselves to help manage their condition, but with little specificity	00
	Physical activity	Willigendael (2004)	48.0%	++
		Owens (2013)	35.0%	++
		Gorely (2015)	Uncertain about benefits	0
	Diet	Willigendael (2004)	32.0%	++
		Owens (2013)	10.0%	+
	Smoking cessation	Willigendael (2004)	30.0%	++
		Owens (2013)	24.0%	+
		Provance (2018)	20.0%	+
Pharmacological Therapy	Cholesterol-lowering and antiplatelet therapy	McDermott (2003)	75.8%	++++

	Medication treatment	Willigendael (2004)	12.0%	+
		Owens (2013)	4.0%	+
	Overall	Martinez (2017)	65.0%	+++

Legend: PAD = peripheral arterial disease; 0 = no knowledge (but % not reported); 00 = some knowledge (but % not reported); + = 0-25% of participants reported knowing about this topic; ++ = 26-50% of participants reported knowing about this topic; +++ = 51-75% of participants reported knowing about this topic; ++++ = > 75% of participants reported knowing about this topic
